# Exploring Experiences and Perspectives of Prescribed Foot Orthoses in People With Diabetes

**DOI:** 10.1002/jfa2.70130

**Published:** 2026-02-02

**Authors:** Niloofar Sedighi, Gordon Hendry, Jari Pallari, Ruth Barn

**Affiliations:** ^1^ Department of Allied Health Professions School of Health and Life Sciences Glasgow Caledonian University Glasgow Scotland; ^2^ School of Engineering Newcastle University Newcastle upon Tyne UK

**Keywords:** adherence, diabetes, focus groups, foot orthoses, foot ulcers, footwear

## Abstract

**Background:**

Adherence to prescribed offloading among people with diabetes is often insufficient. Although factors influencing adherence with prescribed footwear have been well studied, uncertainties remain. Perceptions and views of people with diabetes on their prescribed foot orthoses, including areas for improvement, may offer important insights.

**Methods:**

Using a qualitative study design, a combination of online focus groups and individual interviews were employed to gain a deeper understanding of the experiences and perceptions of people at risk of diabetes‐related foot ulcers regarding experiences of prescribed foot orthoses. All interviews and focus groups were recorded and transcribed verbatim. Transcribed data were coded, and thematic analysis was undertaken following a six‐step thematic analysis framework.

**Findings:**

Eight people with diabetes at risk of developing foot ulcers who had been prescribed foot orthoses were included. Three major themes emerged from thematic analysis: (i) adherence and barriers to effective use of foot orthoses, which captured the role of health professionals as well as the interconnected relationship between footwear and foot orthoses, (ii) perceived benefits of foot orthoses and desired improvements, and (iii) anxiety and psychological impact, which highlighted how anxiety and fear can influence footcare behaviours, both as motivators and barriers.

**Conclusion:**

This study provides valuable insights into user experiences of prescribed foot orthoses and factors influencing foot orthoses–related behavioural choices; these findings may help researchers and healthcare professionals in designing and delivering foot orthoses that better meet the needs of users to enhance engagement with preventative strategies. Important considerations include the perception of balance and stability, the psychological impact of diabetes‐related foot disease and the role of the healthcare professional in providing support both during and after foot orthoses provision.

## 1 Introduction

Foot ulcers are one of the most severe complications of diabetes, impacting both physical and psychological well‐being as well as quality of life [1, 2]. The lifetime incidence of diabetes‐related foot ulcers (DFUs) is estimated at 19%–34%, with recurrence rates of 40%–65% within 1–5 years [3]. Several risk factors contributing to development of a foot ulcer are loss of protective sensation, foot deformity, peripheral artery disease, a history of foot ulceration and any level of lower extremity amputation [4]. Characterised by breaks on the skin of the foot, these ulcers often form due to plantar overload and excessive mechanical stress on the foot of a person with diabetes and loss of protective sensation [5, 6].

Various interventions are recommended to help manage and prevent DFUs including self‐care, education, clinical treatment, and footwear (FW) and foot orthoses (FO) to help offload pressure from high‐risk sites of the foot [4]. A meta‐analysis investigating 18 controlled studies revealed that compared to standard FW/FO, the pressure‐optimised FW/FO are likely to reduce the risk of DFUs in people at high risk of ulceration. However, the effectiveness of these orthotic interventions is heavily influenced by patient adherence [7]. The active engagement of individuals with their foot health and orthotic interventions is essential for the successful prevention of DFUs [8]. Research has shown insufficient adherence to wearing custom‐made FW by individuals with diabetes [9, 10, 11]. Although compliance to prescribed FW has been somewhat studied, little is known about perceptions and experiences of people with diabetes who have been provided with FO. The purpose of this study is to gain a deeper understanding of the views and experiences of people with diabetes on their prescribed FO. The findings of this research may help to inform the future design of FO that better address user needs and preferences.

## 2 Methods

Ethical approval for this study was granted by the Allied Health Ethics Committee, School of Health and Life Sciences, Glasgow Caledonian University (reference number: AHP/A23/023). The study adhered to the regulations specified by the General Data Protection Regulation (GDPR) and the 2018 Data Protection Act.

The study was advertised online through social media posts and through snowball sampling via participants with diabetes who attended a previous patient and a public involvement event and consented to be contacted during future research. People were eligible if they had a clinical diagnosis of diabetes and if they have been prescribed FO for diabetes‐related foot complications, regardless of the FW they used.

A combination of online focus groups (using Microsoft Teams) and one‐on‐one telephone interviews was used in order to engage directly with participants and gather insights into the lived experiences of people living with diabetes who have been prescribed FO. Initially two focus groups were scheduled with four participants in each group, although two participants requested to reschedule. For the two who were unable to attend, one‐on‐one telephone interviews were conducted as an alternative. Each focus group session lasted approximately 60 min, whereas one‐on‐one interviews averaged 25 min. The sessions were conducted by the lead researcher (NS), who comes from an engineering background and therefore had no preconceived ideas regarding FO perceptions or wearing habits. Consequently, their professional background did not influence the data collection or analysis. As a PhD student developing new FO, the researcher was motivated to understand what matters to individuals who use them and remained open to all participant suggestions. At the time of data collection and analysis, the researcher's own FO design features had not yet been started; therefore, this work did not influence the process. The research team also comprised RB and GH, both experienced podiatrists, together with JP, an engineer. Their role in data analysis was to examine and validate the transcripts, codes and themes to maintain accuracy and consensus. NS received training by attending and observing focus groups moderated by GH and by researching and studying best practices for conducting focus groups and followed a semi‐structured format with open‐ended questions, derived from the topic guide (Appendix 1). The topic guide consists of a series of questions and discussion areas. It was initially developed by the research team and later refined through multiple discussions with all members of the research team to ensure alignment with the research objectives. At the start of each session, the researcher (NS) introduced herself as a PhD student conducting the study and outlined the broader DIALECT project, including its aim of developing FO for diabetes‐related foot problems. She explained the objectives of the current study and how participants' contributions could inform future research. Participants were reminded that the session would be audio recorded and transcribed verbatim, and they were asked to reconfirm their consent verbally. At the end of each session, general demographic information including age, history of foot ulcers and self‐reported physical activity levels were captured via an online form. The consolidated criteria for reporting qualitative studies checklist (COREQ) was used for reporting the findings (Appendix 2) [12].

The study was informed by a pragmatic paradigm with a largely realist orientation and adopted a methodological orientation of phenomenology to explore participants' lived experiences [13]. Reflective thematic analysis was employed to identify and describe patterned meanings within participants' accounts, with the aim of generating clinically and design‐relevant insights into adherence and user experience in diabetes FO. A pragmatic approach supports the use of methods that best address applied research aims and produce actionable outcomes. Accordingly, the analytic intent was to describe and interpret participants' accounts as experiential evidence of real‐world barriers and motivators to FO use, with the aim of improving intervention design and clinical care.

All recordings were transcribed verbatim for analysis by the lead researcher, and individual participant IDs were assigned to preserve anonymity. The data were analysed following the six‐phase thematic analysis framework outlined by Braun and Clarke [14]. The data familiarisation phase was undertaken by reading and re‐reading the transcripts to gain an in‐depth understanding of the content. Coding was undertaken by a single coder (NS). Through this process, initial codes were generated by labelling and organising data into groups, related to the research aims. These codes were grouped into superordinate themes and refined through an iterative process of reflection. Subsequently, the initial set of preliminary codes, and themes were then independently reviewed by other members of the research team (RB, GH and JP), who examined the transcripts, codes and themes for coherence, accuracy and alignment with the data. The team discussed their interpretations in multiple consensus meetings during which feedback was incorporated, and as a result, the lead researcher revised the codes and themes accordingly. This iterative process continued until consensus was achieved, and no further modifications were required. Data saturation was monitored throughout the study. No new codes emerged after the final interview. At this point, themes were sufficiently supported across the dataset, and no additional data seemed to change the coding tree. A follow‐up meeting with the research team was held to finalise the themes and ensure consensus on the interpretation of the findings.

## 3 Findings

Eight participants (7 male and 1 female) from Scotland with the average age of 65 years were recruited. Two focus groups were held, and for the two participants unable to attend, individual interviews were conducted over the phone. Characteristics of the included participants and levels of self‐reported FO use and activity levels are summarised in Table 1.

**TABLE 1:** Summary of participants.

Characteristic	Result
Age (mean ± standard deviation)	65 ± 12.6
Sex (male/female)	7/1
Employment (full‐time/part‐time/retired)	4/1/3
History of foot ulcer (yes/no)	3/5
History of amputation (yes/no)	2/6
Number of participants with other diabetes‐related complications	
Foot deformity	3
Heart disease	2
Loss of protective sensation	7
Peripheral artery disease	3
Kidney disease	1
Risk level of participants*	
Low	3
Moderate	2
High	3
Self‐reported use of prescribed foot orthoses	
Not using	1
4–6 h per day	2
More than 6 h per day	5
Types of footwear used with foot orthoses	
Only custom‐made footwear	3
Only off‐the‐shelf shoes	4
Combination of both	1
Use of prescribed foot orthoses at home	
Not using	3
Using	5
Self‐reported physical activity level	
Sedentary (little to no exercise)	2
Lightly active (light exercise or activity 1–3 days/week)	2
Moderately active (moderate exercise or activity 3–5 days/week)	2
Very active (intense exercise or activity 6–7 days/week)	1
Extra active (very intense exercise or a physically demanding job)	1

*Note:* Ulcer risk was assessed according to the IWGDF risk stratification system [15].

Through thematic analysis, NS identified 18 initial codes based on the commonality and differences of experiences. Through an iterative process of reflection, these codes were refined and grouped into 3 superordinate themes that best captured the key themes emerging from the data. More details of the coding tree is provided in Appendix 3. To ensure credibility, two members of the research team (RB and GH) independently reviewed a randomly selected transcript. Consensus meetings were held among members of the research team to review and discuss initial codes and themes to ensure accuracy and reliability of findings. Following these meetings and discussions, the codes and themes were revised and finalised, with a final meeting to confirm the results. The themes identified are as follows: (1) adherence and barriers to effective use of foot orthoses, (2) perceived benefits of foot orthoses and desired improvements and (3) anxiety and psychological impacts.

1. Adherence and barriers to effective use of foot orthoses: This theme summarises the challenges participants faced in consistently using their prescribed FO. Issues such as discomfort, role of FW and the interplay between FO and FW, access to professional support, and timely replacement of FO were cited as important influencing factors. It was clear from the discussions how important the interplay between FO and FW is and how dissatisfaction with prescribed FW can also discourage individuals from using their prescribed FO.

One participant who stopped using their prescribed FO and FW summarised their experience as follows:

P001: ‘*The [custom‐made] boots were the most uncomfortable boots, even though I don’t have much feeling in my feet. They rubbed and cause problems… I can’t use the insoles on ordinary shoes ‘cause they’re too thick. I don’t use the orthopaedic footwear at all, it’s just uncomfortable, it chafes. At the moment, I use Skechers’.*

The compatibility of FO with different types of FW was mentioned both in a negative and a positive way with some participants expressing frustration and others reported positive experiences, noting that their FO fit comfortably across a range of FW as follows:

P002: *‘…the insoles are quite good that they can be transferred between different shoes’.*

Participants had a general understanding of why they were provided with FO and what DFUs are. However, their experiences with the support and care they received varied. On the positive side, some participants expressed satisfaction with their healthcare providers and their services, with one stating

P003: *‘My [healthcare providers] are really good. My team's fantastic!’*.

In contrast, others highlighted experiences of dissatisfaction, with one participant noting

P004: *‘No one’s really given much information to me about what I should be experiencing and not experiencing with these insoles’*.

Another shared their experience, saying

P001: *‘I had an annual diabetic check up on my feet in 2019 and I haven’t had the check‐ups since then’.*

2. Perceived benefits of foot orthoses and desired improvements: This theme highlights how the participants recognised the benefits of the provided FO as well as the areas for improvement. Most participants acknowledged the positive impact of the FO. For instance, one participant stated

P005: ‘*Without the insoles in my shoes, I don't think I would last five minutes’.*

Another person shared a similar experience as follows:

P004: *‘I used to feel like I had little pressure prickles on the bottom of my sole. So [with the insole], that was alleviated…straight away’.*

When asked about the potential areas for improvement, the opinions varied. Some wanted thicker FO for enhanced cushioning and support, whereas others found their FO too thick, causing rubbing and chafing on the dorsal surface of their feet. Some voiced concerns about how the cushioning can affect their foot contact with the ground, explaining that due to neuropathy, too thick and too soft FO make it difficult for them to feel the contact of their foot and the ground and to maintain balance. Some reported incidents of falling and stumbling as a result, citing

P006: *‘If they were made thinner, it’s possible to have more feel and be able to judge when my feet hit the ground, but they wouldn't be as comfortable’.*

P005: *‘When I first got [the insoles], they were too comfortable. I couldn’t feel the ground when I was walking, and I fell over. So, I stopped using them and I went back to the ones that are much less comfortable but actually gave me better contact with the ground’.*

Another participant’s experience was described as

P002: *‘I also have a feeling that I'm walking on a sponge sometimes and I stumble a bit’.*

3. Anxiety and psychological impacts: This theme addresses the psychological burden experienced by individuals with diabetes in relation to managing foot health. Participants expressed concerns about the risk of further complications. Some described how their emotional response towards foot ulceration and amputation including fear and anxiety influences their foot care behaviour. These emotional responses influenced their adherence to recommended foot care practices including the use of FO. In one instance, one participant highlighted,

P004: *‘I do check the soles on my shoes to make sure that there isn’t anything sticking in them because I’m quite prone to standing on tacks and things like that.’*

For some, the perceived severity of their condition was the motivator for consistent use of FO. One participant whose foot condition has declined describes consistency in using FO as follows:

P005: *‘I wear mine [insoles] from the minute I get out of bed in the morning to the minute I go back to it at night… I'm wearing the [insoles] more, but I'm walking less because my disease has advanced to the point where I can't go walking at all.’*

In contrast, another participant explained how they do not view their condition as a severe issue, which leads them to feel less compelled to consistently wear the provided FO and FW as follows:

P007: ‘*I don't have the severity of the problem… obviously it's summer and I would like to wear more casual shoes. Just yesterday I thought we'd have a go at other shoes. Soft shoes. 'Cause I hadn’t gone for soft shoes for a while, and it was nice sunny day.’*

For some individuals, the constant fear of deterioration of their foot condition was significant. One individual who had undergone a minor amputation explained,

P003: *‘Always in the back of my mind I’m worried about anything going wrong with my feet. Anything happening… I’m actually thankful that I’ve still got my foot because they were talking about a complete amputation.’*

Although some learnt to accept their foot condition with a positive outlook, others reported experiencing a loss of social confidence along with disruptions to their lifestyle as follows:

P008: *‘it's kind of embarrassing if you're in a busy place. So, it means that when you go to the supermarket, places like that… I tend to go in when there's nobody else around the car park, because it takes a while to even get out of the car to find your footing’.*

## 4 Discussion

With a move towards personalised medicine and a focus on targeted interventions, there are opportunities to improve adherence to prescribed FO and FW. Despite imaging and technological advances in FO design and manufacture [16], user preferences regarding FO are rarely studied beyond initial comfort levels. Furthermore, adherence to FO and FW is highly variable in diabetes with differing adherence levels observed in and out of the home [10]. In line with the principles of realistic medicine whereby the focus is on aligning care to personal preferences, it is important to consider the views of the individual when implementing treatment plans [17]. Our results indicate mixed levels of satisfaction and adherence to wearing FO, with some individuals abandoning them altogether. Similar experiences have been reported in rheumatoid arthritis with prescribed FW being consigned to ‘shoes in the cupboard’ [18]. Adherence is further complicated in diabetes with the absence of pain due to neuropathy, which may act as a motivator to adherence in other conditions.

During the discussions, it was evident that participants who received both prescribed FW and FO perceived them as interconnected; this is unsurprising because they are frequently provided simultaneously and have similar therapeutic objectives. Whilst everyone in our study was provided with FO for their diabetes‐related foot disease, there were varying levels of deformity, ulcer and amputation experience present. In the UK, FO may be provided by podiatrists, orthotists and physiotherapists and practice varies among professions [19] whereas FW is provided only by orthotists. In our study, dissatisfaction with the prescribed FW often led participants to also discard the FO, partly because the FO were not compatible with standard shoes. Our data highlight the interaction of FO with other interventions, which has previously been reported as often overlooked and not clearly recognised in FO practice models [20].

Functional FO aim to alter the mechanics of the foot whereas accommodative FO provide cushioning and offloading [21]; all types of devices may be prescribed across the spectrum of foot disease in diabetes with bespoke FW indicated where significant deformity exists. The research effort on FO to date focuses on ‘biological endpoints’ such as pressure reduction and kinematics, yet there is uncertainty over their relationship with clinical outcomes [20]. A conceptual model for FO practice has been proposed that incorporates 6 key areas including biopsychosocial factors [20]. Moreover, by a systematic review, it was not possible to find a predictor of adherence to wearing prescribed FW in people with diabetes at high risk of ulceration, and it was suggested that further work should focus on investigating other predictors such as psychological variables [22]. It is clear that we do not yet fully understand adherence to FO in diabetes; our findings indicate that individual perspectives and user experiences may be important.

Another aspect emerging from the themes that influenced adherence, either as a barrier or motivator, is the role of healthcare providers and the quality of care participants received. Although some participants felt ‘very well taken care of,’ others described feeling ‘lost and left alone,’ mainly due to long and delayed check‐ups and limited foot care education. Where there was a perceived lack of support, this negatively affected participants' confidence and overall quality of care. This finding aligns with previous research highlighting the effect of motivation, psychological support and education in accepting assistive devices [22, 23].

Although the positive impact of using FO was largely recognised among participants, their views varied regarding the optimal thickness of the devices. Although cushioning was recognised as essential for better comfort, increased thickness was associated with instability, imbalance and increased risk of falls. Contemporary approaches to FO using additive manufacturing afford opportunities to distribute stiffness properties using a variety of lattice structures to individual areas of the foot [24]. Combining this with individual preferences regarding overall stability characteristics may enhance user experience of the device with the potential to impact on adherence. Given the typically lower levels of physical activity (< 5000 steps/day) and increased mobility challenges reported as diabetes progresses, ensuring that the FO are suitable for each user and useable for normal daily activities is important in improving adherence [25, 26]. Additionally, there seems to be a lack of short‐term follow‐ups after the FO are initially provided to check if any revisions or adjustments are required; this is of course challenging due to the pressures on healthcare providers. However, if people are not using the FO, this may increase future healthcare costs as foot complications are not being prevented.

Consistent with previous research, psychological factors play an important role in the levels of adherence to foot self‐care recommendations [8, 27]. Studies on therapeutic FW have shown that adherence depends on whether individuals view their foot condition as a significant problem and believe that therapeutic FW can solve the problem better than not using them [22]. Similarly, our study found that self‐perception of the severity of the condition influences foot care and adherence behaviour. Fear and anxiety about potential future complications served as both a motivator and a barrier to adherence where some individuals were encouraged to follow foot care recommendations, whereas for others, it led to emotional distress, leading to disengagement and reduced adherence.

## 5 Strengths and Limitations

This study explored experiences and views of prescribed FO in diabetes. Although this study offers valuable insights, certain limitations must be acknowledged. Participants in this study were recruited from across Scotland; therefore, findings may not be transferable to other countries. Furthermore, the FO and FW prescribed to the participants were not standardised. However, it has been reported previously that FO prescription practices vary across professional and international boundaries [19, 21]; thus, standardisation is unlikely to be possible. Additionally, despite technological advances, orthoses provision remains craft based [16] and reliant on clinical decision‐making to inform FO prescription [21]. The variation in FOs may have directly impacted participants' experiences. Finally, consistent with qualitative research, the sample size is small, so further investigation is warranted to explore FO experiences in other populations outside of the UK. Additionally, due to time constraints, transcripts and themes were not returned to participants for member checking, and this is acknowledged as a limitation of this study.

## 6 Conclusion

Investing in preventive measures and effective management strategies such as personalised FO is essential in reducing further healthcare costs associated with diabetes‐related foot complications. Adherence to intervention is a major determinant of treatment success, and understanding user perspectives is key to improving outcomes. We found mixed levels of satisfaction and adherence to prescribed FO. Important elements emerging from the thematic analysis were the perception of stability and balance to ensure confidence to undertake activities of daily living, the interplay of FO with other interventions, and psychological impact of fear and anxiety around diabetes foot complications. Our findings also suggest that the role of the healthcare professional is important in terms of education and support both during and after provision of FO. This study provides valuable insights into user experiences of prescribed FO and factors influencing FO‐related behavioural choices; these findings may help researchers and healthcare professionals in designing and delivering FO that better meet the needs of users to enhance engagement with preventative strategies.

## Author Contributions

**Niloofar Sedighi:** conceptualization, data curation, formal analysis, writing – original draft, writing – review and editing. **Gordon Hendry:** supervision, validation, writing – review and editing. **Jari Pallari:** supervision, writing – review and editing. **Ruth Barn:** conceptualization, supervision, validation, writing – review and editing.

## Funding

This work was co‐funded by UK Research and Innovation (UKRI) under the UK government’s Horizon Europe funding Guarantee Scheme for MSCA Doctoral Networks 2021, the Diabetes Lower Extremity Complications Research and Training Network in Foot Ulcer and Amputation Prevention (DIALECT), RC Grant reference: EP/X02699X/1, and Grant agreement number: 101073533. The funder had no role in the design or conduct of the study.

## Ethics Statement

Ethical approval was obtained from Glasgow Caledonian University, School of Health and Life Sciences Research Ethics Committee (Reference: AHP/A23/023).

## Consent

All participants provided informed consent prior to entry to the study.

## Conflicts of Interest

Dr Gordon Hendry is an associate editor for the Journal of Foot and Ankle Research.

## Data Availability

Data are available on request.

## Appendix 1 Topic Guide

### Researcher Prompts:

- Introduction
- Overview of topic and rationale for focus groups
- Establish that there are no right or wrong answers
- Approximate duration
- Reminder of anonymity, confidentiality and ability to withdraw at any time
- Reminder that conversations will be audio recorded
- Confirm understanding and allow for questions and clarifications
- Confirm consent
- Begin by giving rundown of structure: two main parts to the discussion, going to begin by finding out more about wearing insoles with diabetes and wearing habits of insoles and then ask in more detail about what is important to you in terms of the insole features/design.

### Part 1: Experiences of Wearing Insoles and Wearing Habits

1. Can you describe your experiences of wearing insoles for diabetes‐related foot problems?
  • Are they comfortable? Do they fit inside your shoes?
2. Can you tell me about your understanding of why you have been asked to wear the insoles?
  • Is it related to your foot ulcer(s)?
3. Can you tell me about the kind of footwear you wear during a typical day?
  • Do the insoles fit inside those shoes? Or do you wear shoes provided by an orthotist?
4. Can you tell me how long you normally wear your insoles; do you wear them at all times during all activities? Or do you wear them some of the time?
  • Do you wear the insoles when you are inside your home? Why is that/why not?
5. I wonder if you can think of a place or time when you did not wear your insoles? Why was that?
  • Can you tell me more about why you chose not to wear them?
6. Are there specific activities during which you find the insoles particularly helpful or unhelpful?
7. Has wearing the insoles made a difference to your feet?
  • Do they affect your walking abilities? Balance?
8. Do you feel that the insoles are helping to manage your foot condition?

### Part 2: Important Features of Insoles

9. Is there anything else you can think of that might influence your decision whether or not to wear your insoles?
10. If it was up to you to design your own insoles, would you make any changes?
  • If yes, what would you change and why. If no, what is it you like or find helpful about the insoles?
  • Is there any change that would make the insoles better for you?
11. We are looking at designing new insoles using computer‐aided design and 3D printing to create personalised insoles. We are exploring different materials and design approaches. If I were to list some of the ideas we are considering, I would be interested to hear what you think.
  • We are also interested in reducing waste and being more conscious of the environment; we understand that many insoles are not worn, and we are exploring ways to reduce waste. Is that something that is important to you?
  • How important are the appearance and the feel of the insoles to you?
    - Does the type of material matter; for example, does it matter if it is made from softer or firmer materials?
    - How important is the shape of the insole?
    - Is the colour important at all?
  • Some of the materials are heavier than others. What do you think about the weight of the insoles you have been given?
    - Are you aware of how heavy or light they are? Is it important to you?
  • In terms of comfort, do you have sensation in your feet? How do you decide if something is comfortable?
  • With current ways of making insoles, they often take a while to produce. With 3D printing and computer aided design, we can produce them quicker. Is that something you think is important?
    - If so, why or why not?

Do you have any other comments on insoles?

Thank you for your time and participating in this research.

### Other Areas to Address During the Session:

- Experience with therapeutic insole use
- Types of footwear used with the therapeutic insole
- Diabetes‐related conditions (neuropathy, deformities, history of ulceration and duration of diabetes)
- Nondiabetes‐related musculoskeletal conditions that can affect mobility (arthritis and tendonitis)
- Perceived benefits in ulcer prevention
- Comfort and fit
- Effect on stability and gait
- Usability (for different environments)
- Appearance and style
- Weight
- Reducing pain
- Durability
- Cost

## Appendix 2 COREQ Checklist

**Table:**

Item	Guide questions/description	Study
Domain 1: Research team and reflexivity		
Personal characteristics		
Interviewer/facilitator	Which author/s conducted the interview on focus groups?	Online focus group interviews were conducted by Niloofar Sedighi
Credentials	What were the researcher's credentials? *E.g. PhD, MD*	NS: PhD student; RB: PhD, GH: PhD and JP: PhD
Occupation	What was their occupation at the time of the study?	NS: PhD student; RB: senior lecturer in podiatry, GH: Reader in musculoskeletal health and JP: visiting prof of practice
Gender	Was the researcher male or female?	Female
Experience and training	What experience or training did the researcher have?	NS received training by attending and observing focus groups moderated by GH and by researching and studying best practices for conducting focus groups. GH and RB are experienced in qualitative research.
Relationship with participants		
Relationship established	Was a relationship established prior to study commencement?	Researcher and participants were only in communication briefly via email during the recruitment stage
Participant knowledge of the interviewer	What did the participants know about the researcher? For *example, personal goals and reasons for doing the research*	At the start of the focus group, the researcher (NS) introduced herself as a PhD student, conducting the research; described the wider scope of her project (DIALECT) and the objectives of the present study; and highlighted how participants' contributions could support future research.
Interviewer characteristics	What characteristics were reported about the interviewer/facilitator? For *example, bias, assumptions, and reasons and interests in the research topic*	It was communicated that NS is the lead researcher and a PhD candidate on a larger project (DIALECT) working on developing an insole for diabetes‐related foot problems. The rest of the research team were introduced by their research background and experience; however, they were not present during the sessions.
Domain 2: Study design		
Theoretical framework		
Methodological orientation and theory	What methodological orientation was stated to underpin the study? For example, *grounded theory, discourse analysis, ethnography, phenomenology and content analysis*	Phenomenology
Sampling	How were participants selected? For *example, Purposive, convenience, consecutive and snowball*	The study was advertised via social media in addition to snowball as well as purposive sampling via a network of people who attended a previous event and consented to be contacted.
Method of approach	How were participants approached? For *example, face‐to‐face, telephone, mail and email*	Interested people contacted NS in response to social media posts. Alternatively, potential participants who met the criteria and had consented to be contacted were either contacted by phone or email depending on personal preference.
Sample size	How many participants were in the study?	8 participants were recruited overall
Nonparticipation	How many people refused to participate or dropped out? Reasons?	32 individuals agreed to be contacted; however, 13 were excluded either due to not meeting the inclusion criteria or not wanting to participate. 11 stopped responding to emails and as a result were excluded. In the end, 8 individuals were recruited.
Setting		
Setting of data collection	Where was the data collected? For *example, home, clinic and workplace*	Workplace. A private room was booked at GCU, and the sessions were held through Microsoft Teams or for the ones with conflicting schedules; one‐on‐one telephone interviews were held using NS office telephone.
Presence of nonparticipants	Was anyone else present besides the participants and researchers?	No
Description of sample	What are the important characteristics of the sample? For *example, demographic data and date*	See Table 1
Data collection		
Interview guide	Were questions, prompts and guides provided by the authors? Was it pilot tested?	NS and RB constructed the topic guide with questions for the focus groups and interviews. The guidelines consisted of open‐ended questions based on the areas that were important for the goals of the larger project.
Repeat interviews	Were repeat interviews carried out? If yes, how many?	No, each session and interview were held once for each participant
Audio/visual recording	Did the research use audio or visual recording to collect the data?	Audio recording
Field notes	Were field notes made during and/or after the interview on focus groups?	During the interviews on focus groups and immediately after the sessions, important notes were written down.
Duration	What was the duration of the interviews or focus group?	60–75 min
Data saturation	Was data saturation discussed?	Yes, data saturation was monitored throughout the study. No new codes emerged after the final interview. At this point, themes were sufficiently supported across the dataset, and no additional data seemed to change the coding tree
Transcripts returned	Were transcripts returned to participants for comment and/or correction?	No
Domain 3: Analysis and findings		
Data analysis		
Number of data coders	How many data coders coded the data?	The data were coded by NS
Description of coding tree	Did authors provide a description of the coding tree?	Yes, this information is provided in Appendix 3
Derivation of themes	Were themes identified in advance or derived from the data?	Some themes were identified right after the sessions, and others were identified during the data analysis
Software	What software, if applicable, was used to manage the data?	Microsoft Word
Participant checking	Did participants provide feedback on the findings?	No
Reporting		
Quotations presented	Were participant quotations presented to illustrate the themes/findings? Was each quotation identified? For *example, the participant number*	Yes, each quote is linked to the participants (through participant IDs)
Data and findings consistent	Was there consistency between the data presented and the findings?	Yes, there were difference of opinions and experiences between participants in some instances.
Clarity of major themes	Were major themes clearly presented in the findings?	Yes, major themes were developed based on the codes and were linked to the related codes.
Clarity of minor themes	Is there a description of diverse cases or discussion of minor themes?	All of the initial themes, minor or major, were discussed within the research team, and the major ones were selected as the result of those discussions.
